# Anthropogenic Gradients Drive Divergent Bacterial Communities and C/N/P/S Cycling Functional Gene Profiles in Urbanized Coastal Sediments

**DOI:** 10.3390/microorganisms14102350

**Published:** 2026-10-14

**Authors:** Shuangfei Li, Hongmei He, Wenxuan Zhang, Liao Ouyang, Shuhua Liu

**Affiliations:** 1 Shenzhen Key Laboratory of Marine Bioresource & Eco-Environmental Sciences, College of Life Sciences and Oceanography, Shenzhen University, Shenzhen 518071, China; sfli@szu.edu (S.L.); 2410173031@mails.szu.edu.cn (H.H.); 2 School of Materials and Environmental Engineering, Shenzhen Polytechnic University, Shenzhen 518055, China; wenxuan2727@163.com; 3 State Key Laboratory of Water Resources and Hydropower Engineering Science, Wuhan University, Wuhan 430072, China; shliu@whu.edu.cn

**Keywords:** coastal sediments, bacterial community, QMEC, biogeochemical functional genes, anthropogenic gradients

## Abstract

Coastal sediment bacteria mediate essential biogeochemical cycles, yet their functional gene potential to anthropogenic stress gradients remains insufficiently understood. This study presents a comparative analysis of sediment bacterial communities and carbon, nitrogen, phosphorus, and sulfur (C/N/P/S) cycling genes between the west and east coasts of Shenzhen, China, a region exemplifying sharp contrasts between intensive urbanization and relatively pristine marine environments. We integrated 16S rRNA gene high-throughput sequencing with QMEC (Quantitative Microbial Element Cycling), a high-throughput quantitative PCR chip to profile both bacterial taxonomic composition and quantitatively measured gene abundances across C/N/P/S biogeochemical pathways. Western sediments, under strong terrestrial input, exhibited higher ammonium and organic carbon, while eastern sediments had higher salinity. Bacterial alpha diversity was significantly higher in the west, and community structure differed markedly between regions (ANOSIM R = 0.334, *p* = 0.001). Although dominant phyla (Pseudomonadota, Bacteroidota, and Planctomycetota) were shared, genus-level taxa diverged: *Sulfurovum* (sulfur cycling) was enriched in the west, while organic-degrading *Burkholderia-Caballeronia-Paraburkholderia* and *Phaeodactylibacter* prevailed in the east. Notably, functional gene abundance for all four biogeochemical cycles was an order of magnitude higher in western sediments, with denitrification genes dominating there and nitrification genes in the east. Redundancy analysis (RDA) and the Mantel test identified nitrate as the primary environmental driver of bacterial variation (*r* = 0.574, *p* = 0.001), alongside salinity, temperature, ammonium, and total organic carbon. These findings reveal distinct bacterial adaptations to contrasting anthropogenic pressures and demonstrate that sediment bacterial functional gene profiles can serve as sensitive indicators of ecosystem health, providing a quantitative microbiological basis for targeted pollution management in urbanized coastal zones.

## 1. Introduction

The coastal zone is a key ecotone connecting land and sea. It features extremely high productivity and biodiversity, but it also bears tremendous pressure from land-based inputs and human activities [1,2,3]. Sediment bacteria are the primary drivers of biogeochemical cycles in the coastal zone, controlling essential processes such as the decomposition of organic matter, the transformation of nutrients, and the degradation of pollutants. They also drive biogeochemical cycles of elements such as carbon (C), nitrogen (N), phosphorus (P), and sulfur (S) [4,5,6]. The composition and function of bacterial communities are sensitive indicators of environmental changes [7]. While 16S rRNA gene high-throughput sequencing effectively reveals bacterial community structure and diversity under different stressors [8], taxonomic profiling alone cannot directly infer functional potential [8,9]. Integrating 16S rRNA sequencing with QMEC (Quantitative Microbial Element Cycling) technology offers a robust approach to assess bacterial functional gene potential.

QMEC, a high-throughput qPCR-based functional gene chip, simultaneously quantifies dozens of key genes involved in C, N, P, and S cycling, thus directly evaluating bacterial genetic potential and element turnover capabilities [10,11,12]. It has been successfully applied in agricultural soils [10,13,14], freshwater wetlands [15,16], and sewage treatment systems, effectively capturing bacterial functional gene potential to disturbances such as fertilization, flooding, and pollutant loading. However, systematic QMEC-based investigations remain scarce in coastal sediments, as coastal areas are complex ecosystems affected by land–sea interactions and intense anthropogenic pressure. Previous coastal studies have largely relied on single-gene or single-cycle approaches. For example, qPCR detection of the *amo*A gene [17,18,19,20,21] or *nirS/nos*Z genes [22] to assess nitrification or denitrification potential, and the *dsr*B gene for sulfate reduction processes [23,24]. These studies have provided valuable insights into individual biogeochemical processes; however, the joint distribution of C, N, P, and S cycling-gene abundances, considered together with bacterial community structure, has received comparatively less attention under contrasting eutrophication and salinity conditions. In this study, we combined 16S rRNA gene sequencing with QMEC profiling to address this question in Shenzhen coastal sediments, focusing on co-variation patterns among elemental cycles (e.g., denitrification- and sulfate-reduction-related genes) [25,26] rather than direct functional interactions.

Moreover, QMEC enables a more comprehensive view of the nitrogen cycle—a key process in coastal zones. Beyond conventional markers like *amo*A of ammonia-oxidizing bacteria (AOB) and ammonia-oxidizing archaea (AOA) [18,21], emerging evidence highlights the importance of genes associated with complete ammonia oxidizers (Comammox, e.g., comammox *amo*A) [19,27,28,29] and anaerobic ammonia oxidizers (Anammox, e.g., *hzs*B) [30] in marine sediment nitrogen loss. The QMEC chip includes targets for these novel pathways, allowing us to simultaneously profile both traditional and newly recognized nitrogen transformation potentials in sediments, thereby providing a more complete assessment of nitrogen removal capacity. Understanding how sediment bacteria respond to contrasting anthropogenic pressures has direct implications for coastal management. Functional gene profiles can indicate the self-purification capacity and pollution transformation potential of sediments, helping to identify vulnerable zones and support targeted management. In urbanized coastal regions that experienced different levels of eutrophication and anthropogenic input, such data provide a quantitative microbiological basis for nutrient reduction, sewage control, and ecological health assessment.

Shenzhen, a highly urbanized coastal metropolis situated on China’s southern coast, exhibits marked west–east differences in environmental quality. These disparities are attributable to variations in geographical location, developmental trajectory, and the intensity of human interference [31,32]. The west coast, adjacent to the urban core, receives substantial riverine input, industrial effluents, and domestic sewage, leading to significant nutrient accumulation and organic pollution [33,34]. The east coast, dominated by port operations, tourism, and aquaculture, maintains relatively better water quality but is still impacted by seasonal tourism and coastal development [32,35]. These environmental gradients not only affect aquatic organisms but also profoundly shape sediment bacterial ecosystems [36,37].

In this study, we collected sediments from four representative bays in Shenzhen. By combining environmental parameter measurements, 16S rRNA gene sequencing, and QMEC technology, we aimed to: (i) compare bacterial community diversity and composition between east and west coasts; (ii) elucidate differences in functional gene potentials for C, N, P, and S cycling; and (iii) identify key environmental factors driving shifts in bacterial community structure and function.

## 2. Materials and Methods

### 2.1. Research Area and Sample Collection

**Figure 1:**
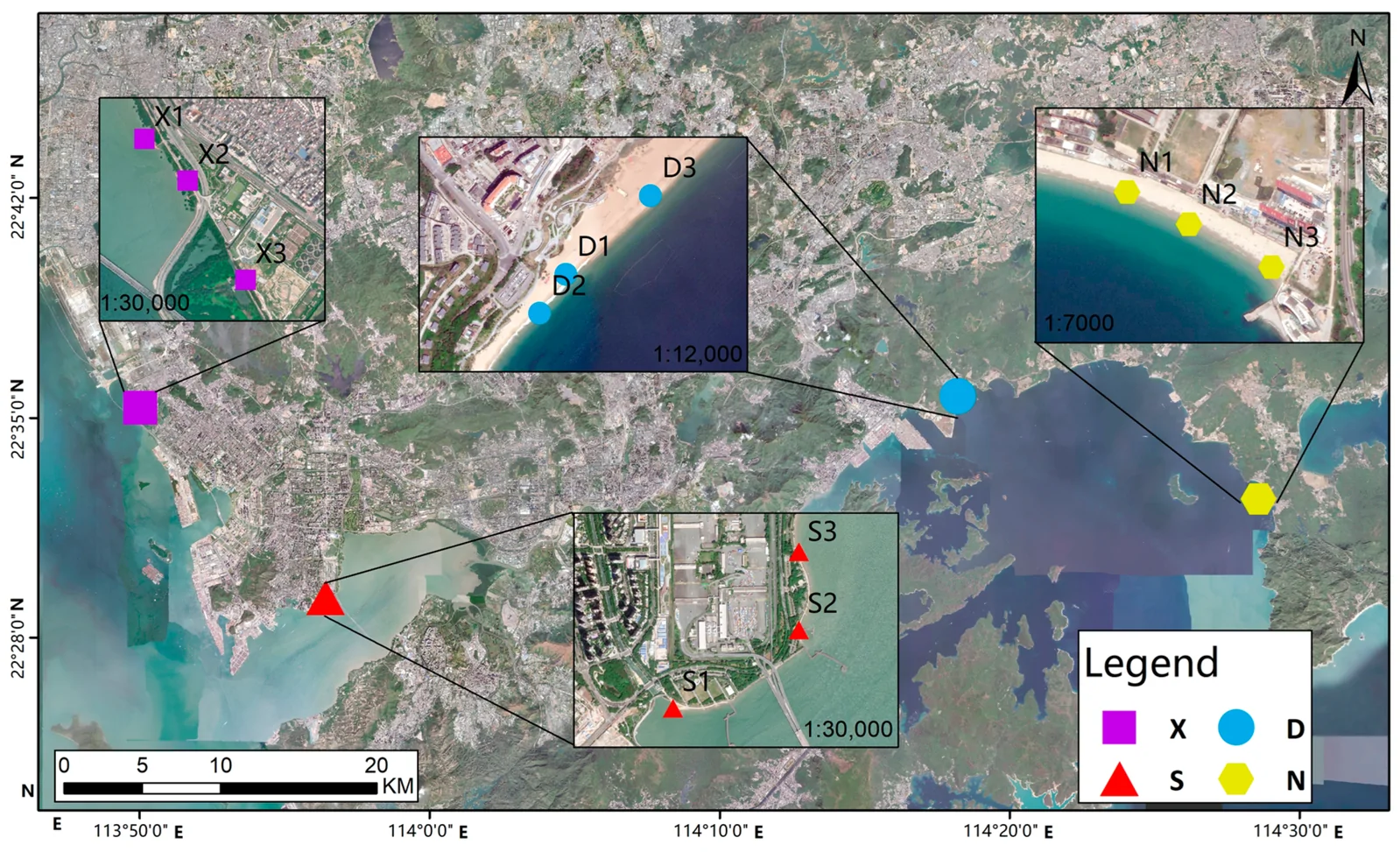
Location of the sampling stations in Shenzhen, China (S, Shenzhen Bay (west); X, Xi Bay (west); D, Dameisha (east); N, Nan’ao (east). Purple, blue, red, and yellow symbols represent X, D, S, and N sites, respectively. Three replicate sampling sites (S1–S3, X1–X3, D1–D3, N1–N3) were collected in each coastal zone.

The sea area of Shenzhen is divided into east and west sea areas based on its geographical location, with a total coastline of 257 km and a sea area of 1400 km^2^. The west coast, including Shenzhen Bay and Xi Bay, is adjacent to the urban core and receives substantial riverine input from the Pearl River, industrial effluents, and domestic sewage, resulting in lower salinity and higher nutrient and organic matter loads [33,34]. The east coast, including Dameisha and Nan’ao, is mainly influenced by port operations, tourism, and aquaculture, and maintains relatively higher salinity and better water quality, although it is still affected by seasonal tourism and coastal development [32,35]. These contrasting environmental conditions make the two regions suitable for examining bacterial community and functional gene responses to different anthropogenic pressures. Four representative coastal zones were selected: Shenzhen Bay (S) and Xi Bay (X) in the west, and Dameisha (D) and Nan’ao (N) in the east [35]. Three repeated sampling sites (S1–S3, X1–X3, D1–D3, N1–N3) were selected in each coastal zone (Figure 1). Surface sediment samples (0–5 cm) were collected using a grab-type sediment sampler and placed in sterile sealed bags. Water depth was within the range of 20–30 cm when sampling. Water samples were collected using a water sampler and placed in sampling bottles [1,32]. All samples were kept on ice and immediately transported to the laboratory. The sediment and water samples were stored at 4 °C, and a subset of each sediment sample was preserved at −80 °C for subsequent molecular analyses.

### 2.2. Determination of Physiological Parameters and Environmental Factors

The physical properties of the water samples were determined using the multi-parameter water quality analyzer (Orion Star A329, Thermo Scientific, Waltham, MA, USA). The concentrations of ammonia nitrogen (NH_4_^+^-N), nitrate nitrogen (NO_3_^−^-N), nitrite nitrogen (NO_2_^−^-N), and available phosphorus (PO_4_^3−^-P) were measured using a fully automated discrete chemical analyzer (CleverChem 380Plus, DeChem-Tech. GmbH, Hamburg, Germany). The analyses were performed following the standard operating procedures and pre-programmed methods provided by the manufacturer.

### 2.3. 16S rRNA Sequencing

Total DNA was extracted from sediment samples using the PowerFecal^TM^ DNA Isolation Kit, and the purity and concentration of DNA were measured using a Nanodrop One (Thermo Fisher Scientific, Waltham, MA, USA). PCR amplification of the V3–V4 region of the bacterial 16S rDNA gene was performed using the universal primers 338F (5′-ACTCCTACGGGAGCAGCA-3′) and 806R (5′-GGACTACHVGGGTWTCTCATAT-3′) [38,39]. The amplification products were purified and then subjected to paired-end sequencing on the Illumina NovaSeq 6000 platform. After the raw sequences were quality-filtered using fastp (v0.14.1) and concatenated using FLASH (1.2.11), the sequences were clustered into operational taxonomic units (OTUs) at a 97% similarity level using UPARSE (v8.1). Taxonomic annotation was performed against the SILVA database version 138.1 (released in 2020), and chloroplast and mitochondrial sequences were removed [40,41,42,43].

### 2.4. QMEC

QMEC (Quantitative Microbial Element Cycling) is a high-throughput qPCR-based technology for quantifying bacterial functional genes, commonly used in environmental bacterial ecology research to evaluate the genetic potential of C, N, P, S, and methane metabolism [11]. The functional genes of the C, N, P, and S cycles in sediments were analyzed by QMEC. The chip contained 71 pairs of validated functional gene primers and one pair of 16S rRNA gene internal reference primers (72 primer pairs in total), covering 35 C-cycling genes, 22 N-cycling genes, 9 P-cycling genes, and 5 S-cycling genes [10]. Amplification was performed using the WaferGen SmartChip real-time PCR system. PCR amplification conditions were as follows: 98 °C for 1 min, 30 cycles of 98 °C for 10 s, 50 °C for 30 s, and 72 °C for 30 s, followed by 5 min extension at 72 °C with three replicates per sample set. The amplification specificity was verified through melting curve analysis. The absolute abundance of each functional gene was calculated based on the absolute copy number of the 16S rRNA gene [11], and the Z-score standardization method was used to compare the differences in the abundance of functional genes among different samples. For 16S rRNA gene sequencing, 36 sediment samples were analyzed, with three field replicates per site. For QMEC, each sample was run in three technical replicates, and only genes detected in all three replicates were considered positive. The mean value of the three technical replicates was used as the gene abundance in each sample.

### 2.5. Statistical Analysis

Data processing and statistical analysis were conducted using R software (v3.5.2). Alpha diversity indices (Shannon, Chao1, Richness, and PD whole tree) were calculated based on OTU tables. Differences among groups were tested using Student’s *t*-test for two-group comparisons and the Kruskal–Wallis test for multi-group comparisons. Beta diversity analysis was conducted under the ‘vegan’ package based on the Bray–Curtis distance matrix for non-metric multidimensional scaling analysis (NMDS), principal coordinate analysis (PCoA), and cluster analysis, and the significance of differences between groups was tested through similarity analysis (ANOSIM). The abundance data of functional genes were normalized by 16S rRNA gene copy number and then Z-score standardized to compare the expression differences between regions. The correlation between environmental factors and the structure of bacterial communities was analyzed by Mantel test, and the influence of environmental factors on community distribution was visualized by using redundancy analysis (RDA). The correlation between the abundance of functional genes and environmental factors was analyzed by Spearman rank correlation analysis, and a heatmap was generated.

## 3. Results

### 3.1. Environmental Factors and Sediment Physicochemical Properties

The characteristics of the sampling sites are summarized in Table 1. The four representative coastal zones in the east and west revealed significant regional differences. The east coast (sites D, N) demonstrated significantly higher salinity (32.67 ± 0.61 psu) than the west coast (sites S, X) (19.6 ± 3.88 psu; *p* < 0.001). Water temperature ranged from 23.5 °C to 31.2 °C, with the west site S showing the highest value (30.4 ± 0.8 °C). The pH was generally weakly acidic to neutral, but eastern sediments (7.28 ± 0.23) were significantly more alkaline than those from the west (6.68 ± 0.14; *p* < 0.001). Nutrient concentrations also exhibited distinct spatial patterns. Ammonium concentrations were highly heterogeneous among sites. Site X exhibited exceptionally high ammonium levels (19.47–25.90 mg/kg), approximately 10–20 times higher than those at the other sites (0.86–2.24 mg/kg). This site-level enrichment drove the higher mean ammonium concentration in the west, rather than a consistent west–east difference. The differences among sites S, D, and N were relatively small compared with site X. Nitrite concentration in the west (0.16 ± 0.13 mg/kg) was significantly higher than that in the east (0.03 ± 0.03 mg/kg; *p* = 0.039). Nitrate was highest at site D (143.08 ± 7.05 mg/kg). Phosphate showed a patchy distribution, with the highest concentration at site S (336.03 mg/kg) and intermediate concentrations at site N (104.15–108.75 mg/kg), while sites X and D had much lower levels. Organic matter and total nitrogen also differed between regions. Total nitrogen (TN) was significantly higher in the west (750 ± 351 mg/kg) than in the east (344 ± 118 mg/kg; *p* = 0.004), showing marked spatial differentiation. Total organic carbon (TOC) reached extremely high values at site N (41,189 ± 2886 mg/kg) but was relatively low at site S (2822 ± 1147 mg/kg).

### 3.2. Alpha Diversity of Sediment Bacterial Community

Sequencing of 36 sediment samples generated 12,899 to 32,655 valid sequences, which were clustered into 1965 to 4298 OTUs at a 97% similarity level. The rarefaction curves tended to flatten, indicating that the sequencing depth was sufficient to cover the vast majority of bacterial groups and that the sample size met the requirements for subsequent analysis.

**Figure 2:**
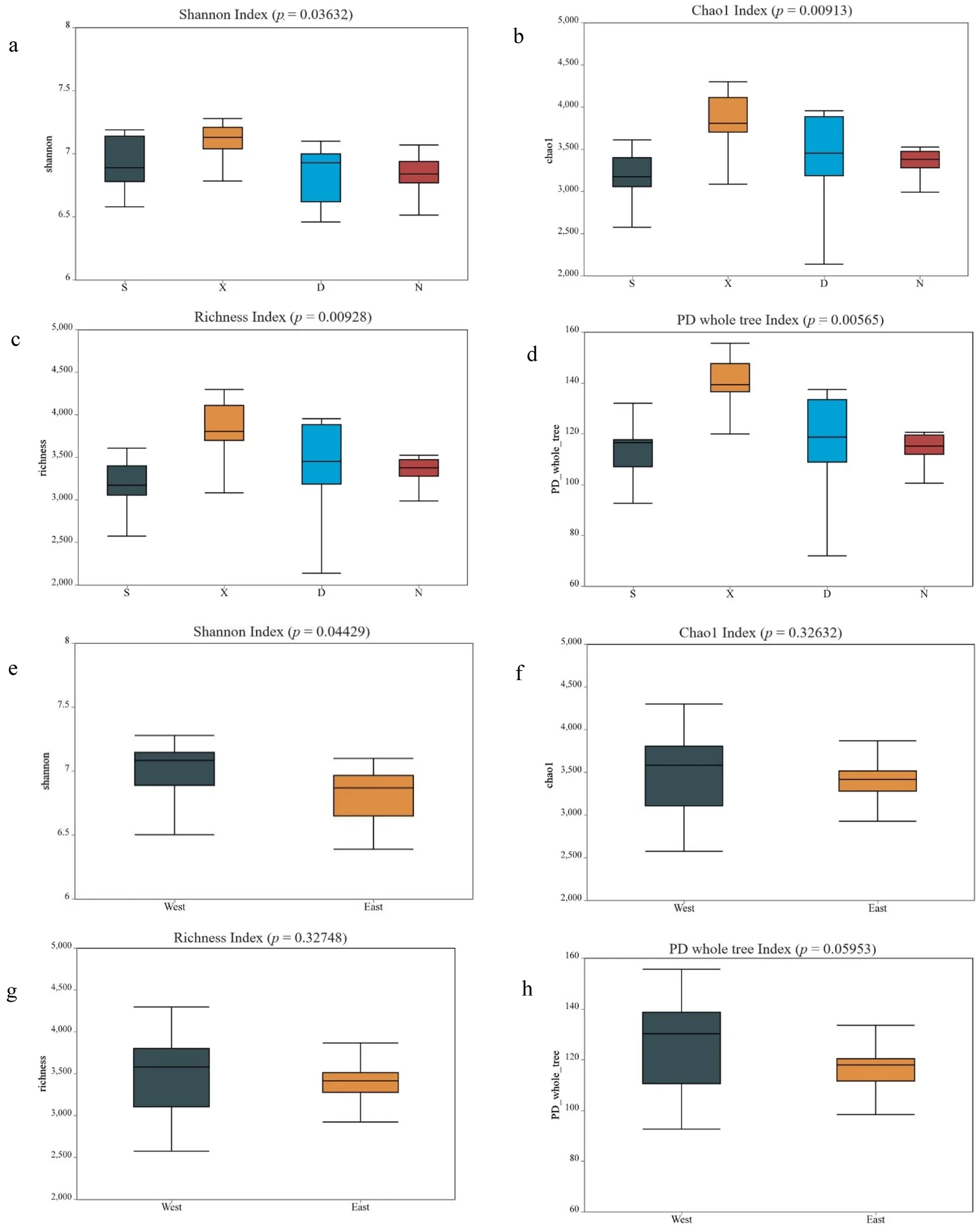
Alpha diversity boxplots of sediment bacterial communities. (**a**–**d**): Sampling sites (S, X, D, N); (**e**–**h**): west (S + X) and east (D + N). Boxes represent the interquartile range (IQR), horizontal lines indicate medians, and whiskers extend to 1.5 × IQR. Differences among the four sites were tested using the Kruskal–Wallis test, and pairwise comparisons were performed using Student’s *t*-test. Significance was set at *p* < 0.05.

Four alpha diversity indices (Shannon, Chao1, Richness, and PD whole tree) were calculated to comprehensively characterize community diversity across sites (Figure 2). All these indices varied significantly among the sampling sites (*p* < 0.05), with ranges of 6.81–7.05 (Shannon), 3185–3811 (Chao1), 3182–3807 (Richness), and 111.42–139.28 (PD whole tree). Site X consistently exhibited the highest values across all indices, indicating the greatest species richness and phylogenetic diversity among the sediments. Pairwise comparisons showed that Richness and Chao1 were significantly higher at site X than at sites S and N (*p* < 0.05), while no significant differences were found among sites S, D, and N. PD whole tree was significantly higher at site X than at all other sites (*p* < 0.05). In contrast, Shannon diversity did not differ significantly between any pair of sites (*p* > 0.05).

When comparing the west and east as a whole, all four alpha diversity indices were higher in the west, though the difference reached statistical significance only for the Shannon index (*p* = 0.044) (Figure 2). These results indicate that sediment bacterial communities in the west, particularly at site X, had higher richness, evenness, and phylogenetic diversity than those in the east. However, the relationship between this elevated diversity and environmental factors was not statistically tested in this study.

### 3.3. Differences in Bacterial Community Composition Analysis

**Figure 3:**
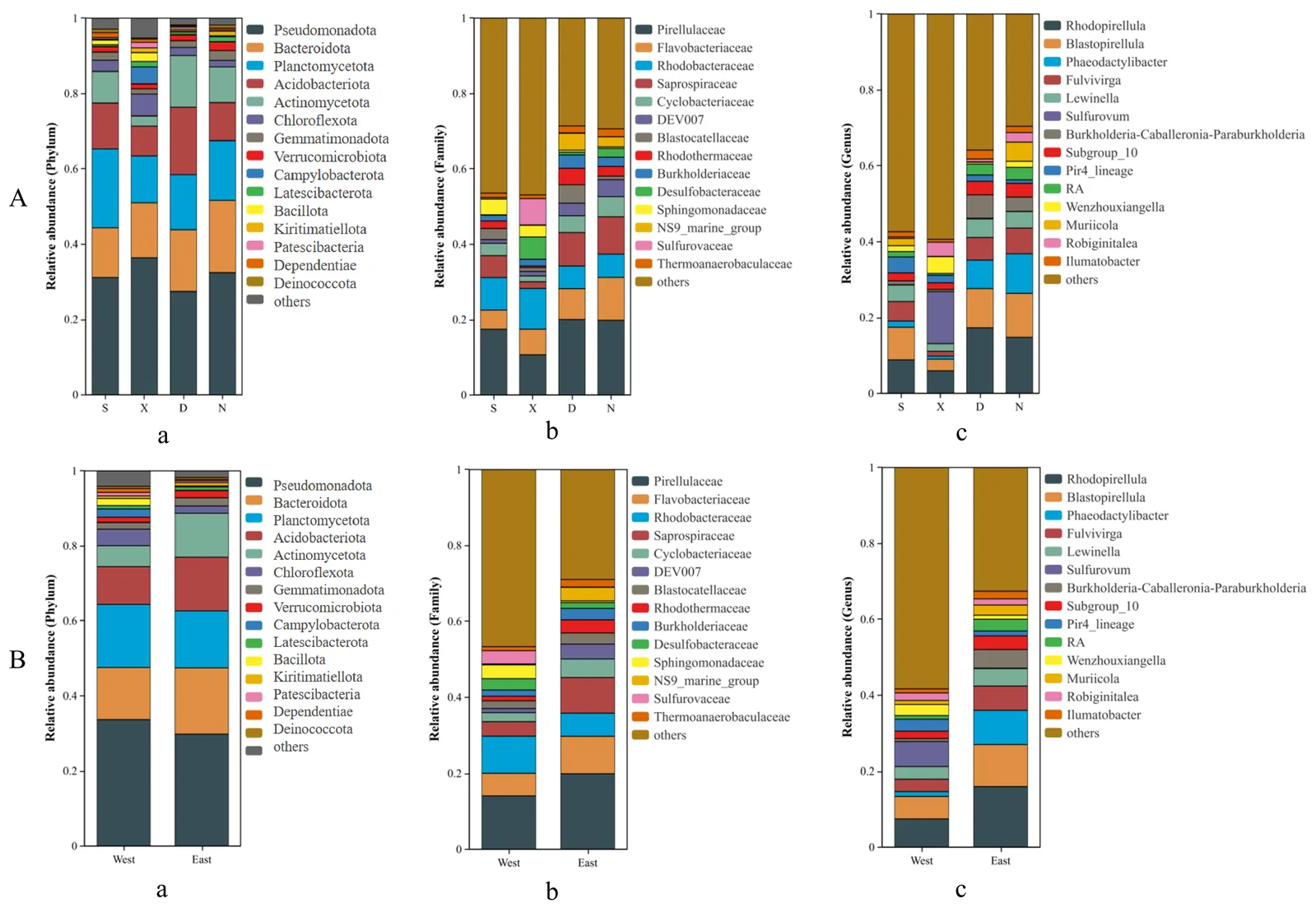
Bacterial community composition of the four sampling points (**A**) and the east and west (**B**) at the phylum (**a**), family (**b**), genus (**c**) levels.

Bacterial community composition was compared among the four sampling sites (sites S, X, D, and N) and between the west and east at phylum, family, and genus levels. At the phylum level, the top four dominant phyla were identical across all sites: Pseudomonadota, Bacteroidota, Planctomycetota, and Acidobacteriota (Figure 3A(a)). The fifth dominant phylum was Actinomycetota at all sites except site X, where Chloroflexota replaced it. Pseudomonadota predominated in all sampling sites, with the highest relative abundance of 36.3% at site X and the lowest abundance of 27.4% at site D. Bacteroidota was the second dominant phylum and ranged from 13.2% (site S) to 19.2% (site N). The highest relative abundance of Planctomycetota was 21.0% at site S, and the lowest was 12.5% at site X. Acidobacteriota ranged from 7.9% (site X) to 17.9% (site D). Chloroflexota was the top five phylum at site X, with a relative abundance of 5.8%. Notably, Campylobacterota—a phylum linked to sulfur cycling—was most abundant at site X (4.5%). When comparing the overall phylum-level composition between the east and west regions (Figure 3B(a)), the relative abundances of Bacteroidota, Acidobacteriota, and Actinomycetota in the east (17.7%, 14.3%, and 11.8%) were higher than in the west (13.9%, 10.1%, and 5.6%). However, the relative abundances of Pseudomonadota and Planctomycetota were slightly higher in the west (33.7% and 16.8%) than in the east (29.7% and 15.2%).

At the family level, marked spatial differentiation was evident (Figure 3A(b)). Pirellulaceae was the most abundant family across all sites, ranging from 10.6% (site X) to 20.0% (site D). Flavobacteriaceae and Saprospiraceae peaked at site N (11.4% and 9.9%, respectively) and were lowest at site X (12.5% and 1.8%, respectively). Rhodobacteraceae was highest at site X (10.8%) and lowest at site D (6.0%). The highest relative abundance of DEV007 was at site N (4.5%), and the lowest was at site S (1.0%). Desulfobacteraceae and Sulfurovaceae were highly enriched at site X (5.9% and 7.0%), which was consistent with the distribution characteristics of Campylobacterota at the phylum level. The relative abundances of dominant bacterial families showed significant differences (Figure 3B(b)). The relative abundance of Rhodobacteraceae in the west (9.7%) was higher than in the east (6.1%). while the relative abundances of Pirellulaceae, Flavobacteriaceae, Saprospiraceae, and Cyclobacteriaceae in the east (19.9%, 9.8%, 9.4% and 4.9%) were higher than in the west (14.1%, 5.9%, 3.8% and 2.4%). Sphingomonadaceae was higher in the west (3.6% vs. 0.5%), but Burkholderiaceae was higher in the east (3.0%) than in the west (1.6%).

At the genus level, *Rhodopirellula* and *Blastopirellula* were among the top five genera at all four sampling sites (Figure 3A(c)). *Rhodopirellula*, *Blastopirellula*, *Phaeodactylibacter*, and *Fulvivirga* all reached the highest relative abundance at site N (14.7%, 11.6%, 10.4% and 6.8%), and the lowest abundances at site X (5.9%, 3.1%, 0.8% and 1.3%). *Lewinella* peaked at site D (4.9%) and was lowest at site X (2.0%). West–east comparisons revealed that all five dominant bacterial genera (*Rhodopirellula*, *Blastopirellula*, *Phaeodactylibacter*, *Fulvivirga*, and *Lewinella*) in the east (16.0%, 11.0%, 9.0%, 6.4% and 4.6%) were higher than in the west (7.4%, 5.9%, 1.2%, 3.3% and 3.2%) (Figure 3B(c)). The most striking difference was *Sulfurovum*, which was nearly absent in the east (0.1%) but reached 6.6% in the west, reinforcing the inference of a more active sulfur cycle in western sediments. Conversely, *Burkholderia-Caballeronia-Paraburkholderia* was more prevalent in the east (4.9% vs. 0.8%).

### 3.4. Differences in Bacterial Beta Diversity

**Figure 4:**
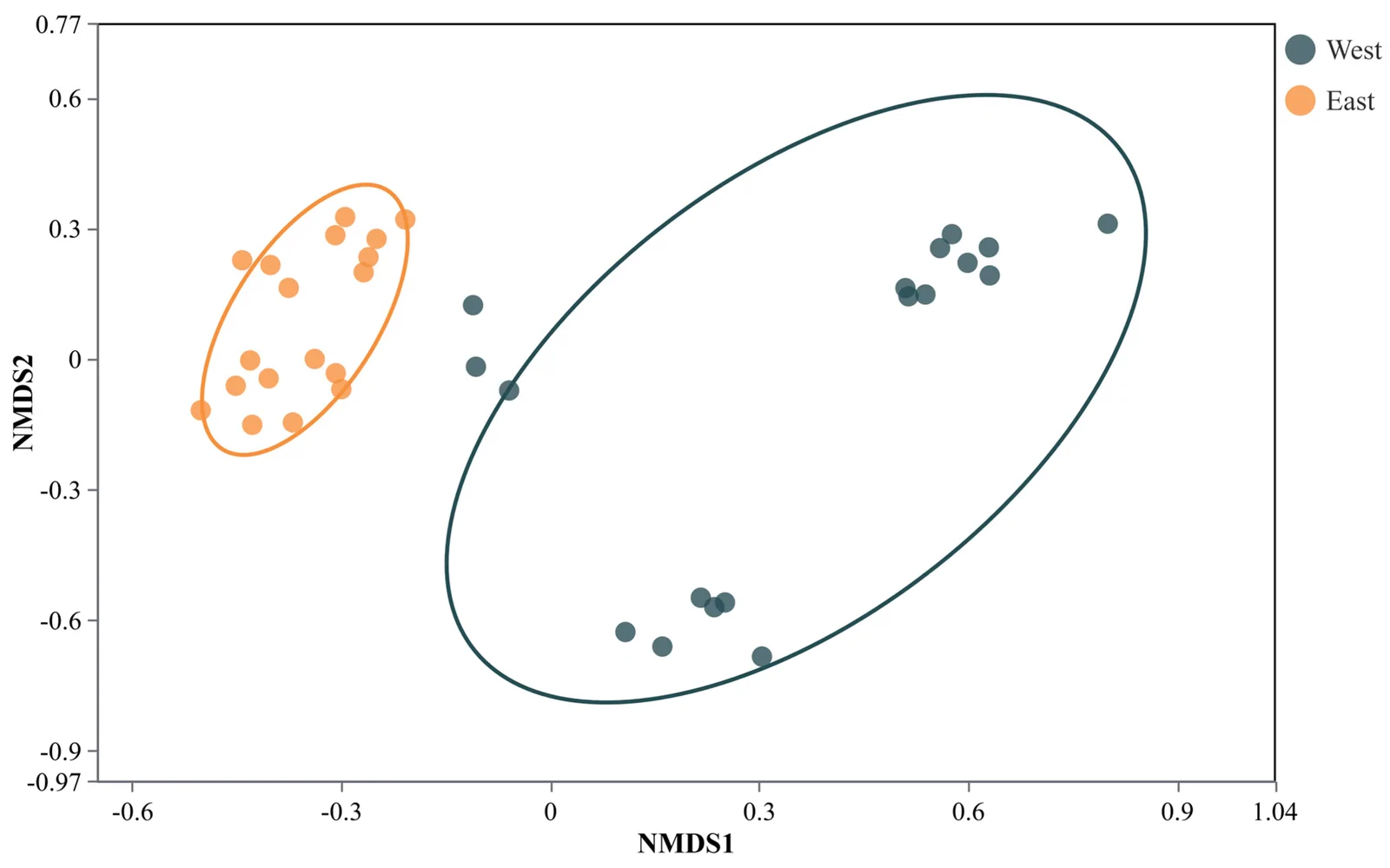
NMDS diagram (Each point represents one individual sediment sample (n = 36). West and east samples are shown in different colors).

To assess spatial variations in bacterial community structure, we performed non-metric multidimensional scaling (NMDS), cluster analysis, and analysis of similarity (ANOSIM) based on Bray–Curtis distance matrices. All three approaches consistently revealed significant west–east differentiation. The NMDS ordination (Figure 4) clearly separated east (site D, N) and west (site S, X) samples into two distinct, nearly non-overlapping clusters. The stress value of 0.084, well below the accepted threshold of 0.2, confirmed that the two-dimensional plot reliably represents the original high-dimensional distance relationships.

ANOSIM analysis further confirmed that the bacterial communities of sediments in the east and west were significantly different (R = 0.334, *p* = 0.001) (Table 2). Within the west, community structures at sites S and X also differed markedly (R = 0.4472, *p* = 0.001). Within the east, sites D and N showed even stronger separation (R = 0.9959, *p* = 0.001). The Cluster Analysis Diagram was also produced (Figure S2); the clear grouping based on west and east confirms that intra-regional community similarity far exceeds inter-regional similarity, consistent with the high ANOSIM R values obtained.

### 3.5. Diversity of Bacterial Functional Genes

To assess the functional potential of sediment bacterial communities, we applied QMEC technology to quantitatively profile key functional genes involved in carbon (C), nitrogen (N), phosphorus (P), sulfur (S), and methane metabolism cycles. Most target genes were successfully detected, with only a few exceptions (exo-chi, *cda*R in C cycle; *hzs*A in N cycle; *cphy* in P cycle) remaining below detection limits.

**Figure 5:**
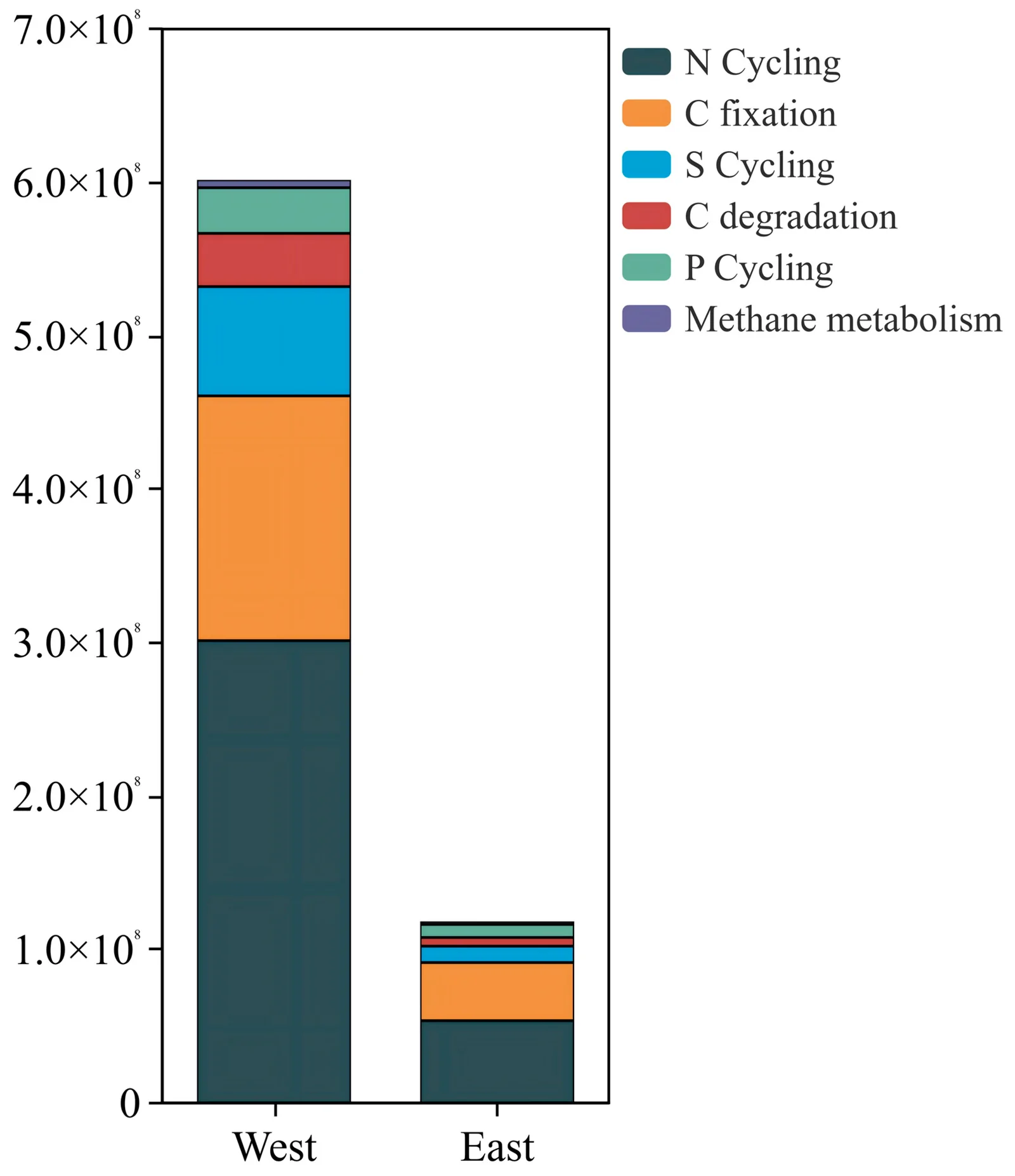
Functional gene stacking diagram (each bar represents the mean value of 18 samples from each region (2 sites × 3 field replicates × 3 technical replicates). Values are expressed as copies per gram of sediment (copies/g)).

The functional gene stacking diagram (Figure 5) illustrates the compositional structure of the six major functional categories in eastern and western sediments. In both regions, N-cycling genes dominated, accounting for the largest proportion, followed by C-fixation and S-cycling genes, while C-degradation, P-cycling, and methane metabolism genes contributed relatively minor fractions. Although the overall composition was broadly similar between the two regions, the absolute abundances of all six gene categories were substantially higher in the west than in the east, often by nearly an order of magnitude (Table 3). Specifically, N-cycling genes were most abundant in both regions, with 3.01 × 10^8^ copies/g in the west and 5.36 × 10^7^ copies/g in the east. The C fixation genes ranked second, with 1.60 × 10^8^ copies/g in the west and 3.79 × 10^7^ copies/g in the east. The abundances of the S cycling, C degradation, and P cycling genes were 7.12 × 10^7^ copies/g, 3.47 × 10^7^ copies/g, 2.98 × 10^7^ copies/g in the west, and 1.07 × 10^7^ copies/g, 5.69 × 10^6^ copies/g, and 8.44 × 10^6^ copies/g in the east. Methane metabolism genes showed the lowest abundance in both regions, with 5.09 × 10^6^ copies/g in the west and 1.97 × 10^6^ copies/g in the east.

Z-score analysis further showed that the vast majority of genes were enriched in the west. Except for a few genes such as *pox*, *amy*A, *nxr*A, and *amo*A2 showing positive Z-values in the east, the other genes showed positive values in the west. On the contrary, only a few genes showed relatively high expression in the east, such as *pox*, *amy*A, *nxr*A and *amo*A2.

### 3.6. Correlation Between Bacterial Communities and Environmental Factors

Mantel tests revealed that most measured environmental factors significantly influenced community composition (Table 4). Nitrate exhibited the strongest correlation (*r* = 0.574, *p* = 0.001), identifying it as the primary chemical driver of beta diversity differentiation. Salinity and temperature also showed a significant positive correlation (*r* = 0.285, *p* = 0.001; *r* = 0.261, *p* = 0.002). In addition, TN, phosphate, TOC, and ammonium were all significantly positively correlated with community structure (*p* < 0.05).

**Figure 6:**
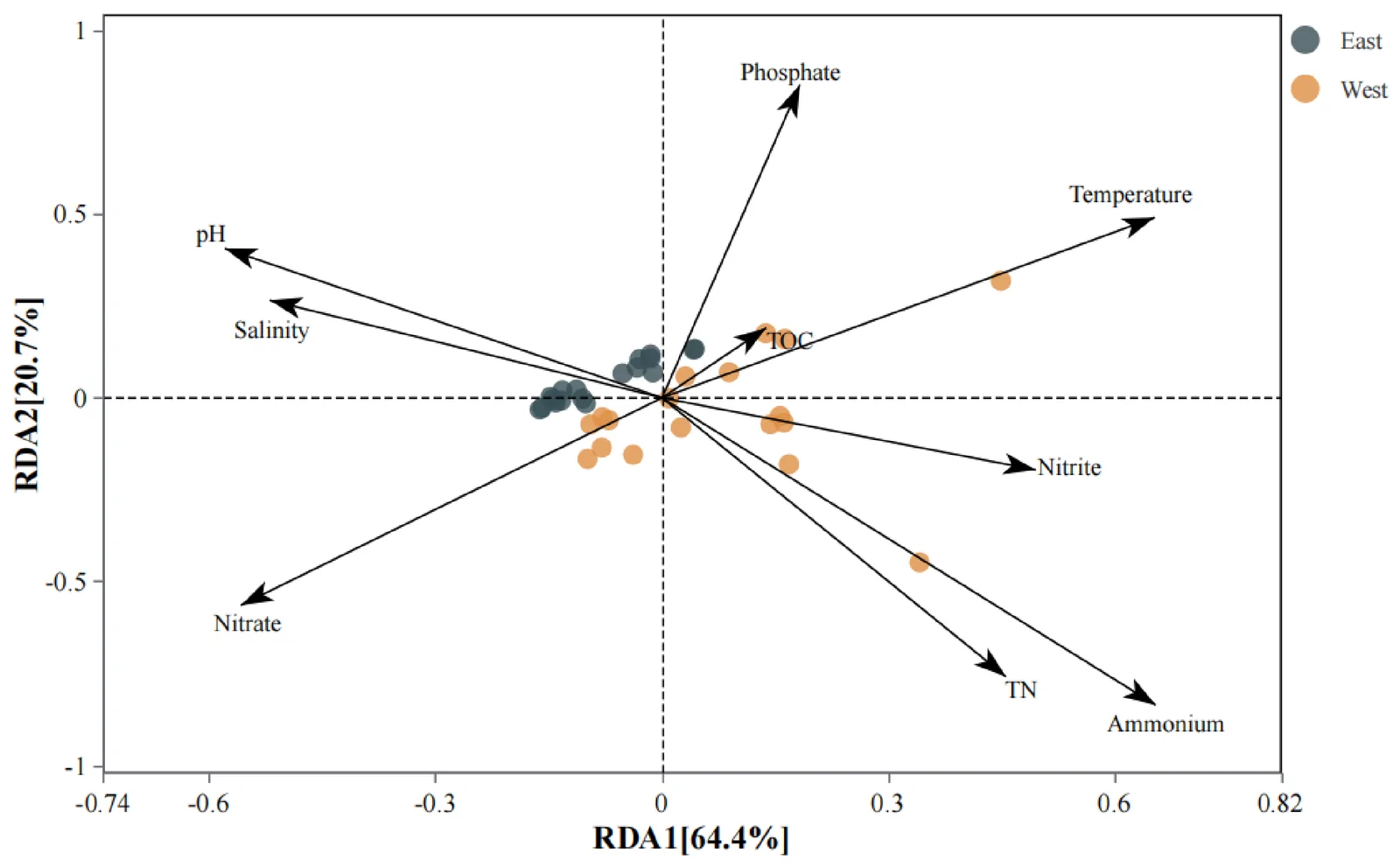
RDA/CCA diagram.

RDA visually demonstrated the influence of key environmental factors on species distribution and corroborated the results of the Mantel test. The first two axes explained 64.4% (RDA1) and 20.7% (RDA2) of the community variation, respectively (Figure 6). Nitrate and ammonium were the most significant gradients shaping species distribution, followed by temperature and phosphate as secondary drivers.

Spearman correlation heatmaps revealed highly specific responses of key genes to environmental factors (Figure S3). For N-cycle genes, multiple denitrification genes (such as *nir*S1, *nir*S2, *nos*Z2) were highly significantly positively correlated with nitrate and TN. The *amo*B was positively correlated with ammonium. For C-cycle genes, C fixation genes (*rbc*L, *acs*A) were positively correlated with salinity and temperature. Certain C degradation genes (*xyl*A, *abf*A) were positively correlated with TOC. For S-cycle genes, sulfate reduction genes (*dsr*A, *dsr*B) were significantly positively correlated with phosphate and salinity. For P-cycle genes, *pho*D was positively correlated with multiple nutrients.

## 4. Discussion

### 4.1. Responses of Bacterial Diversity to Environmental Pollution

The alpha diversity of sediment bacteria in the west was higher than that in the east. In particular, site X exhibited significantly higher values of the Shannon index (7.05), Chao1 index (3811.68), Richness index (3807.56), and PD whole tree index (139.28) than the other sampling sites. This result seemed to contradict the common assumption that pollution leads to a decline in diversity. In this study, the west coast (sites S and X) is mainly affected by domestic sewage, industrial effluents, and riverine input from the Pearl River, leading to high nutrient and organic matter loads [33,34], whereas the east coast (sites D and N) is mainly influenced by port operations, tourism, and aquaculture. Similar phenomena have also been observed in other eutrophic coastal zones [32,35]. For instance, Rubin-Blum et al. reported peak bacterial abundance and production in organic-rich, eutrophic Mediterranean sediments, attributing this to a resource-supply effect whereby nutrient enrichment creates more niches and supports diverse taxa, including pollution-tolerant and degradative groups [44]. This explains our west results: despite high ammonium (20.3 ± 2.63 mg/kg) and organic matter loads (TOC 20,656 ± 3250 mg/kg) at site X, the abundant carbon and energy sources sustain high diversity, consistent with the view that moderate disturbance can alleviate competitive exclusion and promote coexistence [44,45]. The exceptionally high ammonium at site X may reflect intense land-derived nitrogen input.

In contrast, although the east is generally less polluted, it exhibits strong internal heterogeneity. Site D was characterized by high nitrate (143.08 ± 7.05 mg/kg), while site N was characterized by high TOC (41,189 ± 2886 mg/kg). These two contrasting conditions favored specific functional groups, such as the enrichment of nitrification-related groups at site D (*Pir4-lineage*, *Burkholderia-Caballeronia-Paraburkholderia*) and the enrichment of refractory organic matter-utilizing groups at site N (*Lewinella*, *Fulvivirga*, *Sulfurovum*), resulting in decreased overall evenness. Meanwhile, the east was frequently disturbed by tourism and aquaculture activities, which may lead to a simpler community structure. Although there was no significant difference in the Chao1 and Richness indices between the east and the west (*p* > 0.05), the difference in the Shannon index between them was significant (*p* = 0.044), indicating that the difference in species evenness was a more sensitive indicator for distinguishing bacterial diversity between the east and the west. This finding was consistent with the research of Galand et al., who argued that evenness often better reflects community responses to environmental disturbance than richness, as it captures shifts in the balance between dominant and rare taxa [45].

### 4.2. Environmental Drivers of Community Structure Differentiation

Beta diversity analysis confirmed that the community structure of the east and west was significantly separated. NMDS analyses showed that samples from the east and west form two clearly separated clusters (Figure 4). ANOSIM analysis further confirmed that the differences between groups are significant (R = 0.334, *p* = 0.001). ANOSIM analyses in the west and the east both showed extremely significant inter-group differences (R = 0.4472, R = 0.9959 and *p* = 0.001), indicating that the inter-sampling site differences within the region were even greater than the overall differences between the east and west. Within the west, site X was characterized by exceptionally high ammonium (20.3 ± 2.63 mg/kg) and organic matter loads (TOC 20,656 ± 3250 mg/kg), whereas site S had much lower levels. This internal heterogeneity suggests that the west should not be treated as a homogeneous unit, and that site-level environmental gradients play a key role in shaping bacterial community structure. Similar spatial heterogeneity has also been reported in the sediments of the Yangtze River Estuary where within-estuary variation can exceed between-estuary differences [46]. The uneven distribution of TOC suggests that its spatial pattern does not simply mirror overlying water nutrient concentrations but reflects complex heterogeneity in organic matter sources, accumulation, and degradation. The marked separation between site S and site X within the west aligns with the observed gradients in nutrients and organic matter between these two sites.

The Mantel test and RDA further indicated that nitrate was the strongest environmental factor driving community structure differentiation (*r* = 0.574, *p* = 0.001), followed by salinity (*r* = 0.285, *p* = 0.001), temperature (*r* = 0.261, *p* = 0.002), ammonium *(r* = 0.139, *p* = 0.04) and TOC (*r* = 0.148, *p* = 0.02). The ecological significance of nitrate as a key driving factor has been confirmed in multiple coastal zones, including the sediments of Jiaozhou Bay [18]. Nitrate typically originates from anthropogenic inputs (agricultural runoff, wastewater), serving as an indicator of human disturbance [2]. Overall, community differentiation reflects combined effects of natural gradients (salinity, temperature) and anthropogenic nutrient loading, with nitrate as the central link between terrestrial inputs and bacterial shifts. The significant correlations between environmental factors and community structure suggest that freshwater input and spatial temperature gradients both play important roles in community assembly.

### 4.3. Environmental Adaptation Mechanisms of Indicator Bacterial Groups

Bacterial community composition revealed clear west–east differentiation at phylum to genus levels (Figure 3), with indicator taxa distributions closely matching local environmental conditions. The highest relative abundance of *Sulfurovum* was 13.7% at site X, significantly higher than in other sites. *Sulfurovum* was a representative sulfur-oxidizing chemolithoautotroph, initially discovered in extreme habitats such as deep-sea hydrothermal vents [47], and later detected in various marine reductive environments [48,49]. In this study, the enrichment of *Sulfurovum* at site X was highly consistent with the high organic matter load (TOC 20,656 ± 3250 mg/kg) and reducing environment. Similar observations in tsunami sediments showed *Sulfurovum* involvement in sulfur oxidation under aerobic exposure [50], likely reflecting periodic redox fluctuations at site X. Meanwhile, the abundance of S-cycle genes in the west (7.12 × 10^7^ copies/g) was much higher than that in the east (1.07 × 10^7^ copies/g), further confirming the activity of the S cycling in the sediments of the west [4]. *Wenzhouxiangella* and *Robiginitalea* were also enriched at site X (4.4% and 3.7%), together shaping its unique bacterial signature. The higher relative abundance of Campylobacterota at site X implies a more reduced sediment environment there.

*Burkholderia-Caballeronia-Paraburkholderia,* known for aromatic compound degradation [51], was more abundant in the east (4.9%, especially at site D with 6.2%) than in the west (0.8%), likely reflecting terrestrial organic pollution inputs. The relative abundance of *Phaeodactylibacter* in the east (9.0%) was significantly higher than in the west (1.2%). This genus was closely related to algal degradation [52], which may be related to the extremely high TOC (41,189 ± 2886 mg/kg) at site N.

*Rhodopirellula* and *Blastopirellula* were among the top genera across all sites, indicating their core status in these coastal sediments. Planctomycetota played an important role in the marine N cycle, and some groups had anaerobic ammonium oxidation capacity [53]. The genome of *Rhodopirellula* contained a large number of sulfatase genes, which were involved in the degradation of complex polysaccharides [54], indicating its potential function in organic matter degradation. In this study, *Rhodopirellula* was more abundant in the east (16.0%) than in the west (7.4%), which may be related to its response to specific organic matter.

The ANOSIM analysis indicated that the bacterial communities of the two sites are almost completely separated. This difference was highly correlated with the environmental characteristics of the two regions. Site D was characterized by high nitrate (143.08 ± 7.05 mg/kg), and relevant nitrate-oxidizing groups were selected. While site N had extremely high TOC (41,189 ± 2886 mg/kg), enriching organic matter-degrading groups such as *Phaeodactylibacter*. This environment-driven selection aligns with findings from Australian coastal sediments, where local conditions predominantly determine community composition [55].

### 4.4. Functional Gene Potential and Environmental Adaptation

QMEC revealed that absolute abundances of all six functional gene categories were orders of magnitude higher in western sediments than in eastern ones. Specifically, N-cycle genes in the west (3.01 × 10^8^ copies/g) were approximately 5.6 times higher than that in the east (5.36 × 10^7^ copies/g), and S-cycle genes in the west (7.12 × 10^7^ copies/g) were approximately 6.7 times higher than those in the east (1.07 × 10^7^ copies/g). C-degrading genes in the west (3.47 × 10^7^ copies/g) were approximately 6.1 times higher than that in the east (5.69 × 10^6^ copies/g) (Table 3). Despite these abundance differences, gene compositional structure remained largely similar between the east and west (Figure 5), suggesting that environmental stress primarily modulates gene abundance rather than diversity. Western sediments thus exhibit greater elemental cycling potential, particularly for nitrogen removal and sulfur transformation, consistent with Zheng et al. [11], who found that abundance changes often outweigh compositional shifts under environmental gradients. The larger reservoir of functional gene resources in the west suggests substantially higher bacterial genetic potential in this region.

The high nutrient content (ammonium 20.3 ± 2.63 mg/kg) and high organic matter load (TOC 20,656 ± 3250 mg/kg) in the west likely support higher bacterial biomass and gene abundance, while complex pollutant mixtures further induce gene abundance [10]. Similar substrate-driven regulation has been observed in agricultural soils, where denitrification gene (*ni*rK, *nir*S, *nos*Z) abundances correlated positively with organic carbon and nitrogen content [56].

Communities in the west were enriched in denitrification genes (*nir*S, *nos*Z), adapted to high nitrogen and reducing conditions; the east communities showed relative enrichment in ammonia oxidation (*amo*A2) and nitrification (*nxr*A) genes, adapted to higher nitrate and oxidized conditions. This pattern aligns with community compositional shifts and reflects overall bacterial adaptation to environmental stress [57]. Z-score analysis further supported this: genes such as *pox* (C degradation), *am*yA (C degradation), *nxr*A (nitrification), and *amo*A2 (ammonia oxidation) had positive Z-score values in the east, indicating more active aerobic nitrification and specific C-degradation pathways there.

At the genus level, the high abundance of *Sulfurovum* at site X (13.7%) closely paralleled the high abundance of S-cycle genes in the west (7.12 × 10^7^ copies/g), implicating *Sulfurovum* as a major contributor to sulfur oxidation, as its genome contains complete sox gene clusters for sulfide/thiosulfate oxidation [58]. This distribution pattern aligns with a recent study in the Beibu Gulf, which also found that sulfur oxidation genes dominated in nearshore sediments, underscoring the critical role of geographical location in shaping sulfur cycling potential [59]. The high abundance (4.9%) of *Burkholderia-Caballeronia-Paraburkholderia* in the east may be linked to the organic pollutant degradation capabilities carried by this group [51].

### 4.5. Distribution Characteristics of Nitrogen-Cycle Genes and Their Environmental Regulation

QMEC analysis revealed that N-cycle genes dominated the gene pool in both eastern and western sediments, confirming coastal zones as hotspots for nitrogen transformation [60]. Their total abundance in the west (3.01 × 10^8^ copies/g) was markedly higher than that in the east (5.36 × 10^7^ copies/g). This result was consistent with the high ammonium (20.3 ± 2.63 mg/kg) and high TN (1289 ± 209 mg/kg) in the west, indicating that the terrestrial nitrogen input directly promoted the functional potential of N-cycling bacteria [61]. Denitrification genes (*nir*S1, *nir*S2, *nos*Z2) were positively correlated with nitrate and TN, aligning with previous findings that denitrification potential increases with nitrogen availability [62]. The high nitrogen load in the west thus provides favorable conditions for denitrification. Conversely, the ammonia oxidation gene (*amo*B) correlated positively with ammonium, suggesting ammonium concentration influences ammonia-oxidizer distribution and activity [63]. Genes *amo*A2 and *nxr*A in the east were relatively enriched (with positive Z-scores), which was consistent with the high nitrate (71.8 ± 42.62 mg/kg) and relatively oxidized environment in the east. However, under extremely high organic matter and nitrogen loading, bacterial interaction may shift from cooperative to competitive, potentially suppressing certain nitrogen-cycling genes such as amoA [64], suggesting that the relationship between nutrient enrichment and gene abundance is not always monotonic. The positive correlations between denitrification genes and nitrate and TN suggest that a high nitrogen load may enhance denitrification potential. The positive correlations between C fixation genes and salinity and temperature may reflect the distribution of photosynthetic or chemoautotrophic bacteria. The positive correlations between sulfate reduction genes and phosphate and salinity suggest possible coupling between sulfur and phosphorus cycling and salinity regimes. The positive correlation between *pho*D and multiple nutrients reflects its responsiveness in nutrient-rich environments.

Overall, the west was dominated by denitrification (nitrogen removal), while the east was dominated by nitrification (nitrogen transformation)—a strategy differentiation that aligns with regional environmental traits: high ammonium and reducing conditions in the west favor denitrifiers, whereas high nitrate and oxidizing conditions in the east support nitrifiers. This trade-off reflects community-level functional adaptation to environmental stress [65]. The enrichment of denitrification genes in the west coincided with the high abundance of facultative anaerobic groups like *Sulfurovum*, some strains of which (e.g., *Sulfurovum denitrificans*) possess complete nitrate reduction capacity, suggesting potential sulfur-oxidation-denitrification coupling [66]. In the east, the relative dominance of ammonia oxidation/nitrification genes may relate to nitrifying taxa such as *Nitrospira* and *Nitrosomona*s [67], providing a basis for predicting functional potential from community composition. In summary, nitrate was the strongest physicochemical driver of bacterial spatial differentiation in Shenzhen’s coastal sediments, acting in concert with salinity and temperature.

Notably, Comammox *Nitrospira*—capable of completing full nitrification independently [67,68]—was detected in our sediments. Given the contrasting nitrate-rich (east) and ammonium-rich (west) environments, different Comammox lineages may be selected for in each region, warranting further investigation.

## 5. Conclusions

This study reveals clear environmental gradients and distinct bacterial characteristics between the eastern and western sediments of Shenzhen. Western sediments—particularly site X—exhibited higher bacterial diversity, enhanced C, N, P, and S cycling potential, and were dominated by sulfur-oxidizing bacteria and denitrification processes. In contrast, eastern sediments showed higher salinity, enrichment of organic-degrading and nitrification-related functional groups, and a nitrification-dominated nitrogen cycle. Nitrate emerged as the key driver shaping both community structure and functional gene distribution, acting in concert with salinity, temperature, ammonium, and TOC. These findings advance our understanding of bacterial responses to anthropogenic pressure in urbanized coastal zones and provide a scientific basis for ecological health assessment and targeted pollution management strategies.

## Figures and Tables

**Table 1:** Environmental Factors and Physicochemical Properties.

Area	Site	Salinity	Temperature	pH	Ammonium	Nitrite	Nitrate	Phosphate	TN	TOC
		psu	°C		mg/kg	mg/kg	mg/kg	mg/kg	mg/kg	mg/kg
WEST	S1	21.44 ± 0.05	31.2 ± 0.1	6.6 ± 0.03	2.238 ± 0.131	0.089 ± 0.025	0.435 ± 0.197	336.029 ± 136.044	233 ± 33	3200 ± 1604
WEST	S2	17.78 ± 0.02	31.2 ± 0.1	6.9 ± 0.02	1.571 ± 0.011	0.057 ± 0.005	0.453 ± 0.125	74.458 ± 15.502	200 ± 0	1133 ± 549
WEST	S3	6.4 ± 0.16	28.7 ± 0.26	6.9 ± 0.003	2.113 ± 0.12	0.068 ± 0.006	0.43 ± 0.122	51.708 ± 2.413	200 ± 0	4133 ± 176
WEST	X1	22 ± 0	23.7 ± 0.1	6.3 ± 0.03	25.904 ± 0.068	0.651 ± 0.02	103.011 ± 1.203	0.293 ± 0.042	1633 ± 67	26,800 ± 1361
WEST	X2	22.67 ± 0.33	25.7 ± 0.15	6.8 ± 0.02	19.467 ± 0.133	0.044 ± 0.03	65.077 ± 1.645	0.168 ± 0.024	833 ± 33	14,167 ± 590
WEST	X3	27.33 ± 0.33	26.1 ± 0.03	6.5 ± 0.02	15.518 ± 0.333	0.044 ± 0.017	99.737 ± 0.839	0.591 ± 0.062	1400 ± 0	21,000 ± 231
EAST	D1	30.68 ± 0.55	23.7 ± 0.07	6.9 ± 0.003	1.004 ± 0.003	0.022 ± 0.003	129.78 ± 2.853	1.602 ± 0.256	133 ± 67	333 ± 167
EAST	D2	32.88 ± 0.03	23.5 ± 0.09	7 ± 0.03	0.863 ± 0.006	0.016 ± 0.003	142.258 ± 0.308	0.777 ± 0.397	467 ± 33	6033 ± 939
EAST	D3	33.04 ± 0.01	23.5 ± 0.15	7.3 ± 0.04	0.699 ± 0.012	0.151 ± 0.006	157.2 ± 1.406	0.884 ± 0.638	700 ± 58	8500 ± 1041
EAST	N1	33.03 ± 0.52	25.8 ± 0.15	6.9 ± 0.23	2.238 ± 0.037	0.001 ± 0.001	0.518 ± 0.087	108.746 ± 2.696	300 ± 0	46,367 ± 2898
EAST	N2	33.63 ± 0.03	26.2 ± 0.18	7.6 ± 0.06	2.03 ± 0.048	0.013 ± 0.009	0.354 ± 0.167	104.145 ± 2.165	200 ± 0	36,967 ± 726
EAST	N3	32.73 ± 0.07	26.1 ± 0.15	7.9 ± 0.05	2.257 ± 0.051	0.008 ± 0.008	0.709 ± 0.174	106.273 ± 1.898	267 ± 33	40,233 ± 1267

**Table 2:** ANOSIM analysis based on Bray–Curtis distance.

Group	R-Value	*p*-Value
West vs. East	0.3340	0.001
S vs. X	0.4472	0.001
D vs. N	0.9959	0.001

**Table 3:** Absolute Abundance of Bacterial Functional Genes in Eastern and Western Sediments (copies/g).

Class	West (copies/g)	East (copies/g)
N Cycling	3.01 × 10^8^	5.36 × 10^7^
C fixation	1.60 × 10^8^	3.79 × 10^7^
S Cycling	7.12 × 10^7^	1.07 × 10^7^
C degradation	3.47 × 10^7^	5.69 × 10^6^
P Cycling	2.98 × 10^7^	8.44 × 10^6^
Methane metabolism	5.09 × 10^6^	1.97 × 10^6^

Note: Absolute gene abundances were quantified by QMEC high-throughput qPCR and expressed as copies per gram of sediment (copies/g).

**Table 4:** Mantel Experiment on the Influence of Environmental Factors on the composition of Bacterial communities.

Factor	*r*	*p*
Temperature	0.261022782	0.002
pH	−0.045263702	0.741
Salinity	0.284666199	0.001
Ammonium	0.139243096	0.04
Nitrite	−0.026086945	0.558
Nitrate	0.573512834	0.001
Phosphate	0.177980316	0.008
TN	0.182232716	0.016
TOC	0.147834068	0.02

## Data Availability

The original contributions presented in this study are included in the article/Supplementary Materials. Further inquiries can be directed to the corresponding author.

## Supplementary Materials

The following supporting information can be downloaded at: https://www.mdpi.com/article/10.3390/microorganisms14102350/s1, Figure S1. Rarefaction curve of the samples; Figure S2. Relative Abundance of the samples in Genus Level; Figure S3. Spearman correlation heatmap between C/N/P/S cycling gene abundances and environmental factors; Table S1. Sequencing statistics for all sediment samples; Table S2. Number of OTUs and valid sequences for each sediment sample; Table S3. Z-score of individual functional genes involved in carbon (C), nitrogen (N), phosphorus (P), sulfur (S), and methane metabolism in east and west sediments.
