# Supplementary material for: Anthropogenic Gradients Drive Divergent Bacterial Communities and C/N/P/S Cycling Functional Gene Profiles in Urbanized Coastal Sediments
Source: Microorganisms. 2026 Oct 14;14(10):2350. doi: 10.3390/microorganisms14102350 (PMC13707710; doi:10.3390/microorganisms14102350)
Supplement: Supplementary file 1 [file microorganisms-14-02350-s001.zip › microorganisms-4578633-supplementary.pdf]

Supplementary Materials

Anthropogenic Gradients Drive Divergent Bacterial Communities and C/N/P/S Cycling Functional Gene Profiles in Urbanized Coastal Sediments

Shuangfei Li[1], Hongmei He[1,2], Wenxuan Zhang[2], Liao Ouyang[2,*], Shuhua Liu[3]

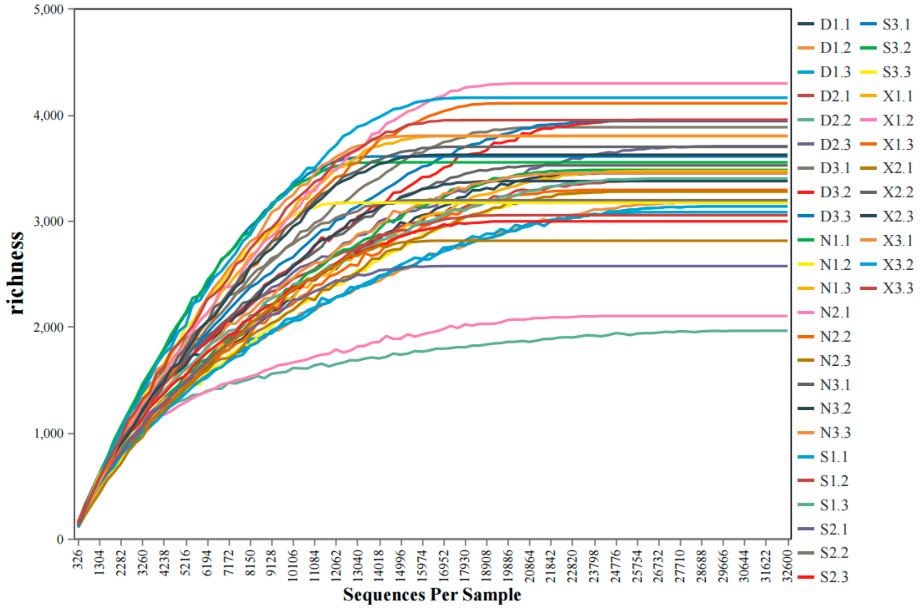

Figure S1. Rarefaction Curve of the samples

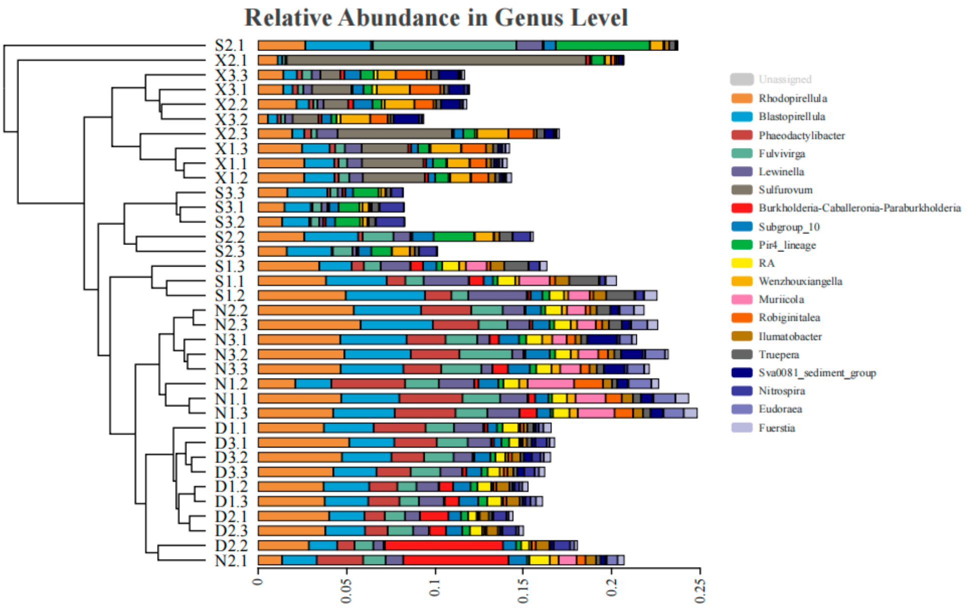

Figure S2. Relative Abundance of the samples in Genus Level

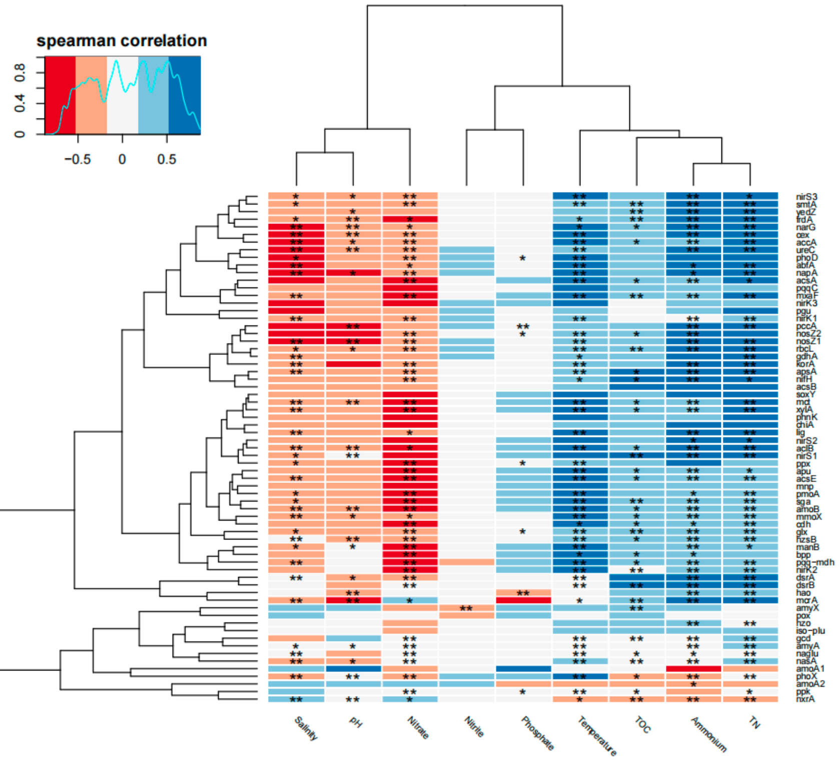

Figure S3. Spearman correlation heatmap between C/N/P/S cycling gene abundances and environmental factors.

Table S1 Sequencing statistics for all sediment samples.

Sample ID	CCS reads	N50	Total Base	AvgLen	AvgQuality
S3.3	35557	1458	51690580	1453.74	40.81
S3.2	39572	1459	57556936	1454.49	41.12
S3.1	38471	1459	55955285	1454.48	41.25
S2.3	37152	1455	53929108	1451.58	41.15
S2.2	38596	1455	56036166	1451.86	41.07
S2.1	39002	1448	56378946	1445.54	41.48
S1.3	37661	1454	54687990	1452.11	41.51
S1.2	38069	1451	55163437	1449.04	41.58
S1.1	38402	1450	55596010	1447.74	41.91
X3.3	37450	1460	54568939	1457.11	41.15
X3.2	37061	1464	54108296	1459.98	41.36
X3.1	38070	1460	55475845	1457.21	41.29
X2.3	36354	1456	52783432	1451.93	41.5
X2.2	38303	1463	55902152	1459.47	41.12
X2.1	36457	1453	53000756	1453.79	41.76
X1.3	36704	1452	53105844	1446.87	41.46
X1.2	39530	1453	57262835	1448.59	41.41
X1.1	36166	1452	52348179	1447.44	41.72
N3.3	36872	1459	53673743	1455.68	41.65
N3.2	37412	1459	54434774	1455.01	41.4
N3.1	36217	1459	52721331	1455.71	41.29
N2.3	38651	1460	56382555	1458.76	41.42
N2.2	37079	1460	54044922	1457.56	41.65
N2.1	30858	1461	45052230	1459.99	41.53
N1.3	37879	1455	55057908	1453.52	41.67
N1.2	37238	1458	54260027	1457.11	41.62
N1.1	36235	1454	52619992	1452.19	41.5
D3.3	36400	1458	53056800	1457.6	41.3
D3.2	37707	1458	54966047	1457.71	41.45
D3.1	36126	1459	52632760	1456.92	41.52
D2.3	38404	1459	55984761	1457.78	41.26
D2.2	37235	1458	54290066	1458.04	41.19
D2.1	33971	1460	49549494	1458.58	41.23
D1.3	37165	1456	54135044	1456.61	41.45
D1.2	37829	1456	55076712	1455.94	41.49
D1.1	39264	1455	57121891	1454.82	41.48

Table S2 Number of OTUs and valid sequences for each sediment sample.

SampleID	No. of OTUs	No. of seqs
D1	2685	26525
D2	3147	28173
D3	3042	22364
X1	2754	16864
X2	2536	15008
X3	3077	14781
S1	2664	23110
S2	2731	15890
S3	2528	11597
N1	2644	23567
N2	2412	23586
N3	2621	19897

Table S3 Z-score of individual functional genes involved in carbon (C), nitrogen (N), phosphorus (P), sulfur (S), and methane metabolism in east and west sediments.

Class	Gene	East	West
C degradation	pox	-0.707	0.707
C degradation	iso-plu	0.707	-0.707
C degradation	apu	0.707	-0.707
C degradation	amyX	0.707	-0.707
C degradation	xylA	0.707	-0.707
C degradation	exo-chi	NA	NA
C degradation	chiA	0.707	-0.707
C degradation	sga	0.707	-0.707
C degradation	naglu	0.707	-0.707
C degradation	mnp	0.707	-0.707
C degradation	manB	0.707	-0.707
C degradation	glx	0.707	-0.707
C degradation	cdh	0.707	-0.707
C degradation	amyA	-0.707	0.707
C degradation	pgu	0.707	-0.707
C degradation	abfA	0.707	-0.707
C degradation	cex	0.707	-0.707
C degradation	lig	0.707	-0.707
C fixation	rbcL	0.707	-0.707
C fixation	mcrA	0.707	-0.707
C fixation	pccA	0.707	-0.707
C fixation	mct	0.707	-0.707
C fixation	acsE	0.707	-0.707
C fixation	acsA	0.707	-0.707
C fixation	accA	0.707	-0.707
C fixation	korA	0.707	-0.707

Class	Gene	East	West
C fixation	cdaR	NA	NA
C fixation	aclB	0.707	-0.707
C fixation	acsB	0.707	-0.707
C fixation	smtA	0.707	-0.707
C fixation	frdA	0.707	-0.707
Methane metabolism	mmoX	0.707	-0.707
Methane metabolism	mxaF	0.707	-0.707
Methane metabolism	pmoA	0.707	-0.707
Methane metabolism	pqq-mdh	0.707	-0.707
N Cycling	nirS3	0.707	-0.707
N Cycling	nosZ2	0.707	-0.707
N Cycling	hzsA	NA	NA
N Cycling	nasA	0.707	-0.707
N Cycling	amoB	0.707	-0.707
N Cycling	nirK3	0.707	-0.707
N Cycling	amoA1	0.707	-0.707
N Cycling	nirK1	0.707	-0.707
N Cycling	napA	0.707	-0.707
N Cycling	hao	0.707	-0.707
N Cycling	gdhA	0.707	-0.707
N Cycling	ureC	0.707	-0.707
N Cycling	hzo	0.707	-0.707
N Cycling	nxrA	-0.707	0.707
N Cycling	nosZ1	0.707	-0.707
N Cycling	amoA2	-0.707	0.707
N Cycling	nirS2	0.707	-0.707
N Cycling	narG	0.707	-0.707
N Cycling	nifH	0.707	-0.707
N Cycling	nirK2	0.707	-0.707
N Cycling	hzsB	0.707	-0.707
N Cycling	nirS1	0.707	-0.707
P Cycling	phoD	0.707	-0.707
P Cycling	bpp	0.707	-0.707
P Cycling	gcd	0.707	-0.707
P Cycling	phoX	0.707	-0.707
P Cycling	ppk	0.707	-0.707
P Cycling	pqqC	0.707	-0.707
P Cycling	ppx	0.707	-0.707
P Cycling	phnK	0.707	-0.707
P Cycling	cphy	NA	NA
S Cycling	dsrA	0.707	-0.707
S Cycling	dsrB	0.707	-0.707
S Cycling	apsA	0.707	-0.707

Class	Gene	East	West
S Cycling	soxY	0.707	-0.707
S Cycling	yedZ	0.707	-0.707

(Note: Z-scores were calculated based on the mean abundance of each functional gene in the east and west groups after normalization to the 16S rRNA gene copy number. Positive and negative values indicate relative enrichment in the corresponding region. "NA" indicates that the gene was below the detection limit. Gene categories follow the QMEC classification.)
